# Autologous Cell Delivery to the Skin-Implant Interface via the Lumen of Percutaneous Devices *in vitro*

**DOI:** 10.3390/jfb1010014

**Published:** 2010-11-25

**Authors:** Antonio Peramo

**Affiliations:** Department of Oral and Maxillofacial Surgery, 1150 W Medical Dr. MSRBII A560, University of Michigan, Ann Arbor, MI 48109, USA; E-Mail: aperamo@umich.edu; Tel.: +1-734-615-8298; Fax: +1-734-763-6199

**Keywords:** biointegration, cell delivery, cell-material interaction

## Abstract

Induced tissue regeneration around percutaneous medical implants could be a useful method to prevent the failure of the medical device, especially when the epidermal seal around the implant is disrupted and the implant must be maintained over a long period of time. In this manuscript, a novel concept and technique is introduced in which autologous keratinocytes were delivered to the interfacial area of a skin-implant using the hollow interior of a fixator pin as a conduit. Full thickness human skin explants discarded from surgeries were cultured at the air-liquid interface and were punctured to fit at the bottom of hollow cylindrical stainless steel fixator pins. Autologous keratinocytes, previously extracted from the same piece of skin and cultured separately, were delivered to the specimens thorough the interior of the hollow pins. The delivered cells survived the process and resembled undifferentiated epithelium, with variations in size and shape. Viability was demonstrated by the lack of morphologic evidence of necrosis or apoptosis. Although the cells did not form organized epithelial structures, differentiation toward a keratinocyte phenotype was evident immunohistochemically. These results suggest that an adaptation of this technique could be useful for the treatment of complications arising from the contact between skin and percutaneous devices *in vivo*.

## 1. Introduction

Percutaneous devices and several other surgical implants are becoming ubiquitous in contemporary medicine. The increased dependence on advanced treatments with technologically improved delivery systems will produce a sustained growth in the number of patients undergoing procedures where a skin-implant interface will be present, e.g., a variety of catheters and long-term intubation systems or osseointegrative prosthetic limb devices, where the prosthesis is permanently integrated with the body [1,2,3]. Several others have been described elsewhere [4]. On the other hand, it is expected that the number of medical procedures with more sophisticated implants and longer periods of implantation will increase in the near future due to increased age population with several medical complications, particularly diabetes and vascular diseases. Increasingly, these biointerfaces will also become long-term or permanent interfaces.

The interface between tissues and medical implants is prone to infections and, over time, is not conducive to the integration of the implant into the tissue, ending with implant failure. Such failures are more common for percutaneous implants due to the permanent disruption to the skin. Conversion of a surgical implant into a dual purpose device allowing the timed, on-demand, repetitive local delivery of cells, regenerative materials, drugs and anti-microbials from the *modified* implant itself to the surrounding tissues can help in reducing implant failure. There are a number of good reasons for the implementation of regenerative strategies around the implants: the possibility that the material or cell suspension will reduce the inflammatory response of the tissue to the device; reduce or eliminate recurrent scarring; eliminate or reduce acellular and avascular areas; and, in particular, the possibility of *inducing tissue regeneration*. The concept is then the construction of a dynamic/active biointerface that permits delivery of regenerative materials, including cells, when regeneration is desired. An extended discussion of the advantages of skin-implant interface regeneration was presented previously [5]. Successfully addressing the problems originating at this biointerface will also be helpful to avoid or reduce complications in surgical procedures that require long-term permanent access to internal areas after total implantation [6,7].

To start addressing these issues with percutaneous devices, we have previously presented three *in vitro* model systems to provide proof-of-concept of the feasibility of delivering materials at the skin-percutaneous device interface [8,9,10], permitting the analysis of the interface between the skin and external devices. Extending that work, here the possibility of delivering autologous cells through the implant itself is briefly described. While the intention of the delivery of biomaterial suspensions were to demonstrate that the technique is feasible and the biomaterials affected the biology of the skin [8], the delivery of the cells is intended as a proof-of-concept that cell delivery around the skin-implant interface is technically possible, as suggested previously [5] and can possibly be used as a regenerative technique for tissue repair around the implant, in a similar way to the use of keratinocytes suspensions for wound repair [11,12].

## 2. Experimental Section

**Figure 1:**
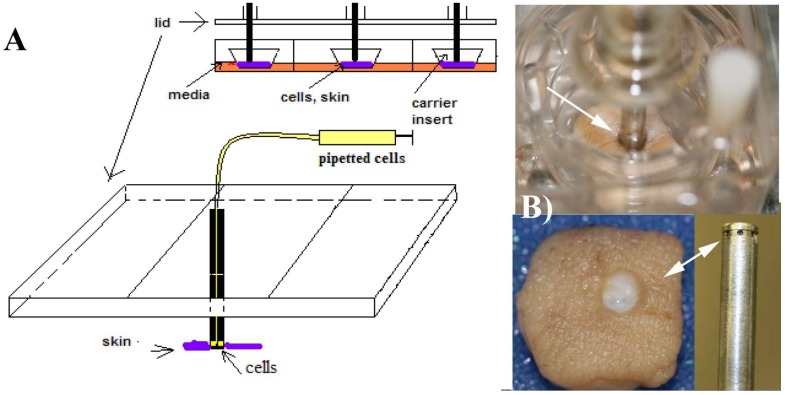
(A) The modified Transwell culture system developed previously [8,9,10], indicating how the cells were delivered to the skin specimens. The skin explants are located at the bottom of the Transwell carriers and perforated with the pins, which have several orifices to deliver the cell suspension. Conceptually, this system represents a soft-tissue anchored percutaneous device; (B) A specimen perforated with a fixator pin is shown on the top of the image. The cells come out of the pin’s orifices and are delivered to the areas around the rim of the skin. The picture was taken after extracting the pin from the skin. In this simplified system, absolute and precise control of the location of the delivery is not possible, but as seen in Figure 2, the cells were located in the dermal areas under the rim.

Human skin was obtained and prepared for the experiments as reported elsewhere [8]. The skin was punctured with a 3 mm diameter sterile biopsy punch. Experiments consisted of a culture plate containing a skin specimen interfaced with a fixator pin, as seen in Figure 1, which shows the experimental system with its components. The medium to culture the skin in the wells was changed daily. After reception of the skin biopsy, a piece was used for extraction and culturing of primary keratinocytes [13]. Skin specimens of approximately 1.5 cm^2^ were cut using a scalpel and cultured for ten days at 37 °C and 5% CO_2_ atmosphere. The specimens were placed epidermal side up at the air-liquid interface in a Transwell culture system (Organogenesis, Canton, MA) modified with a specially designed lid to attach fixator pins. Fixator pins were hollow stainless steel 316, ISO 5832-9 4 mm OD, 3.6 mm ID and 10 cm long, (McMaster-Carr). These hollow pins had six orifices machined at the bottom of the pin for delivery of the cell suspension, as explained next.

After culturing the keratinocytes and the skin specimens separately for eight days, a suspension of keratinocytes was obtained using standard trypsinization techniques and re-suspended in the same culture medium used to culture the skin specimen. This suspension was delivered, using the tip of a pipette, through the top aperture of the pin. This was done in a single shot containing a total of 4 × 10^5^ cells. Separately, from the same stock suspension of keratinocytes used for the delivery, immunocytochemical analysis of the cells was performed. This was done by cytospinning the cell suspension at 750 rpm for 5 minutes and depositing the cells in glass slides. The slides with the cells were fixed in 95% ethanol before performing antibody analysis. Two days later (day 10 of culture), the skin specimen was collected for histological and immunohistochemical analysis, presented below. For histological analysis, biopsies were fixed in 4% phosphate buffered paraformaldehyde for 24 h, routinely dehydrated and paraffin embedded. Serial sections were obtained at 5 μm. For cytospun cells and skin sections, the presence of the epithelial markers involucrin (epidermal cell differentiation), keratin 10 (K10, which is seen in all suprabasal cell layers including stratum corneum) and ki67 (proliferation) was analyzed [14]. Histological evaluation of the skin was performed with hematoxylin and eosin (H&E) and tissue sections were cut from the area adjacent to the perforation where the fixator pin was located. For light microscopy analysis, images were taken using a Nikon E800 microscope (Melville, NY, U.S.).

## 3. Results and Discussion

The purpose of this work was to provide initial evidence of the possibility of using modified percutaneous implants as means for autologous cell delivery to areas in contact with the skin. Cell transplantation has been used as regenerative therapy in various settings [15]. The use of cell transplantation was previously suggested as a possibility for implementation at the skin-implant interface [5]. Additional possibilities are to mix the cells with other products (biomaterials, growth factors, drugs or antimicrobials) to induce biological changes in the affected areas.

The technique presented is new. It is relatively common to deliver cells (autologous or not) *percutaneously* (typically using some type of catheter or injecting the cells using a syringe). However, the technique presented uses a replica of a *percutaneous implant* (the most simple, a fixator pin) that has been modified to allow the delivery of the cells. Percutaneous devices are intended to be implanted for long periods of time, some of them permanently. Their modification will permit the use of this technique on a recurrent basis, if needed.

The use of an *in vitro* system with organotypic cultures of human skin has some limitations, particularly regarding the total time the explants can be cultured. By using autologous cells, the total number of cells that could be injected was limited because they had to be extracted from the same tissue that is cultured as explant, right after surgery. The ten day period used in this experiment was decided solely on the basis of the maintenance of the skin explants, which typically cannot be cultured for much longer. Shorter periods (for example, three or five days) may have been possible, but the limitation would have been the need to rapidly culture the keratinocytes. The experiment was designed to show autologous cell delivery with primary (non-frozen) cells, but it is expected that sufficient amounts of previously frozen cells could also be used. More advanced experiments could possibly be designed to deliver a substantially higher number of cells using a time point strategy.

**Figure 2:**
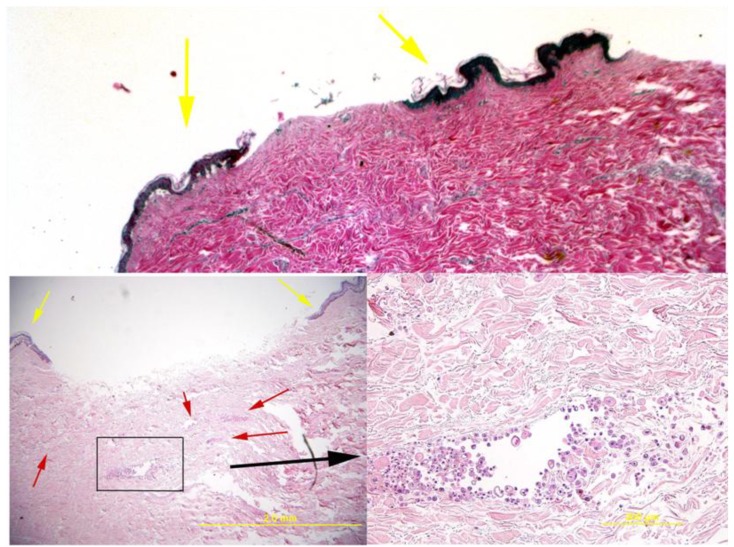
Histology of the skin explant around the pins. The top image shows a control where no cells were delivered and the bottom images show a sample with delivered cells (additional control examples can be observed in [8]). In these images, the delivered cells are apparent as small multifocal aggregates within the dermis (red arrows in bottom left image and the boxed area that is magnified in the right bottom image). Histologically, the cells resembled undifferentiated epithelium and had variations in size and shape. Viability was demonstrated by the lack of morphologic evidence of necrosis or apoptosis. Yellow arrows show the epidermal areas of the skin that formed the rim around the fixator pin.

Histopathological analysis indicated that there was epidermal and dermal autolysis in the skin, which is a normal condition for organotypic cultures of the skin after 10 days of culture [10]. This is particular visible in the epidermal compartment. Figure 2 shows that the delivered cells formed multifocal aggregates perivascularly and interstitially within the dermis. These cells are round to polygonal and have varying amounts of bright pink cytoplasm.

**Figure 3:**
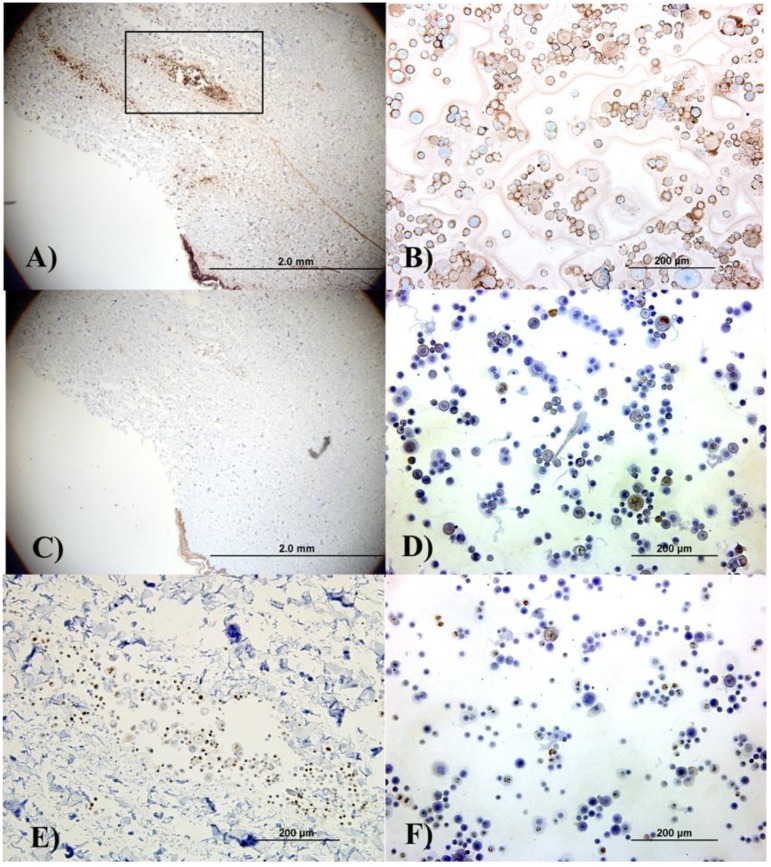
Immunohistochemical analysis of the skin explants with the delivered keratinocytes (Figure A, Figure C and Figure E) and separately cultured keratinocytes (B, D, F) indicates that, although the cells did not form organized epithelial structures in the explants due to insufficient culture time, keratinocyte phenotype was not lost indicating that the delivery process did not affect the cell’s epithelial characteristics. (A) and (B) show involucrin staining; (C) and (D) show keratin 10 staining; and (E) and (F) show ki67 staining. (E) shows the cell aggregate located in the dermal area represented by the black box in (A). Left column pictures are all from sections from the skin explant while right column pictures correspond to autologous cytospun keratinocytes.

In addition to the regular H&E histological characterization, basic analysis of cellular differentiation, cell proliferation and confirmation of keratinocyte phenotype was performed. Involucrin functions as an intermolecular cross-bridge of the keratinocyte cornified envelope and is normally found in the upper spinous layers, but its presence is increased in proliferative or hyperproliferative cells. Figure 3A and Figure 3B show that the keratinocytes within the epidermis, in the aggregates in the skin, and those cytospun from the cell suspension are differentiating. When keratinocytes are proliferative, involucrin would be present in several (if not all) epithelial layers except for the basal layer, given that the epidermis is rapidly differentiating. Proliferation in the cultured and delivered cells was confirmed by the presence of the proliferation marker ki67. This is observed in Figure 3E (an image of the cell aggregate in the dermis) and 3F, which corresponds to cytospun cells. Finally, light staining for keratin 10 (an epithelial marker seen in all suprabasal cell layers including the stratum corneum) was observed in both the skin section and the cytospun cells, (Figure 3C and Figure 3D).

## 4. Conclusions

Three conclusions can be drawn from this experiment: first, qualitatively, it was observed that the cells survived the delivery process; second, autologous cells can be used, even in *in vitro* models in experiments where there is a limitation in the time the tissue explants can be cultured; third, the cells can be delivered by performing adequate modifications of the percutaneous implants. The short culturing period allowed by the use of organotypic cultures of skin did not provide any indication of future activity of the keratinocytes, for example the possibility of forming completely renovated epithelium or scar tissue. Additional possibilities for testing include, for example, the delivery of fibroblasts or a combination of cells or use of specially designed percutaneous devices before implementing this concept in animal models. In this respect, it is not clear in what conditions the *in vivo* results may differ from the *in vitro* results. For instance, in small animal studies, transplanted cardiomyocytes integrated into damaged heart [16], but in a porcine model most of the transplanted cells were separated from host cardiomyocytes by scar tissue [17]. Complementary and combination therapies with other materials or specific molecules that reduce scar formation, *i.e.,* growth factors [18] may be necessary.
